# Orthorexic tendencies are linked with difficulties with emotion identification and regulation

**DOI:** 10.1186/s40337-020-00291-7

**Published:** 2020-04-23

**Authors:** L. Vuillier, S. Robertson, M. Greville-Harris

**Affiliations:** Faculty of Science and Technology, Department of Psychology, Bournemouth University, Poole, UK

**Keywords:** Orthorexia nervosa, Alexithymia, Emotion dysregulation, Eating disorders

## Abstract

**Background:**

Orthorexia nervosa (ON) is characterised by an unhealthy obsession with healthy eating and while it is not recognised as an eating disorder (or any disorder), current research is exploring similarities and differences with such disorders. The literature has shown that individuals with eating disorders have difficulties identifying and describing emotions (known as alexithymia) as well as regulating them. However no research to date has looked at whether people with orthorexic tendencies also suffer from difficulties with emotions. In this paper, we refer to people with orthorexic tendencies but do not assume that their healthy eating is at a pathological level needing clinical attention.

**Methods:**

The current study examined this by asking 196 healthy adults with an interest in healthy eating to complete four questionnaires to measure ON (ORTO-15 – reduced to ORTO-7CS), eating psychopathology (EAT-26), alexithymia (TAS-20) and emotion dysregulation (DERS-16).

**Results:**

We found that difficulties identifying and regulating emotions was associated with symptoms of ON, similar to what is found in other eating disorders. We suggest that ON behaviours may be used as a coping strategy in order to feel in control in these participants who have poor emotion regulation abilities.

**Conclusions:**

Our results show that individuals with ON tendencies may share similar difficulties with emotions compared to other eating disorders. While important, our results are limited by the way we measured ON behaviours and we recommend that further research replicate our findings once a better and more specific tool is developed and validated to screen for ON characteristics more accurately.

## Plain English summary

Orthorexia nervosa (ON) is characterised by an unhealthy obsession with healthy eating. While it is not a recognised eating disorder (or a recognised disorder altogether) it seems to share many similarities with them, including an unhealthy obsession around food and feelings of guilt over food transgression. While individuals with eating disorders have been shown to have difficulties identifying and describing their feelings (known as alexithymia) as well as regulating their emotions, this has not been shown in people with symptoms of ON. Our research with 196 participants revealed an association between emotion identification and regulation, and symptoms of ON. This suggests that individuals with orthorexic tendencies may have difficulties identifying as well as regulating their emotions, such as resisting impulse and finding the right strategies to decrease the intensity of their emotions when upset, which is similar to what is found in other eating disorders. We suggest that ON behaviours may be used as a coping strategy in order to feel in control. While important, our results are limited by the way we measured ON symptoms. Indeed, the scale used in this study measured our participants’ tendency towards an obsession with healthy eating but did not measure whether their healthy eating was at a pathological level or needing clinical attention. We recommend that further research replicate our findings once a better and more specific tool to measure ON is developed and validated to more accurately screen for ON tendencies.

## Background

Orthorexia Nervosa (ON) was defined in 1997 by Bratman [1] as a fixation on healthy eating, with “*Ortho*” meaning righteous and “*Orexia*” meaning appetite. Despite increasing media attention, it has not yet been recognised in any diagnostic manual such as the Diagnostic and Statistical Manual of Mental Disorders (DSM-5) [2]. Research surrounding ON is notably limited [3], and while there are conflicting opinions surrounding the definitive diagnostic criteria for ON, making identifying the prevalence an on-going challenge, it seems that around 1% of the general population may be affected [4]. While ON is thought to be different from avoidance/restrictive food intake disorder (ARFID) [5] in that it is an obsession around eating *clean* food, rather than food that have certain sensory properties^1^, it has been found to have similarities with anorexia nervosa (AN) and bulimia nervosa (BN) [6], and has even been suggested to develop in the recovery phase of an eating disorder [7]. However, more research is needed to identify whether ON is similar to, or different from, other disorders so we can best understand and eventually treat it. A potential aspect to consider is whether emotions play a role in the development of maintenance of ON symptoms. Indeed, emotions, particularly difficulties identifying and regulating emotions have been widely reported in the eating disorder literature [8–10]. The current study addresses this gap by looking at whether ON symptoms are also linked with difficulties with emotions.

### Note 1:

Someone with ARFID might be avoiding and/or restricting their intake for a number of different reasons, including sensory properties of the food, but also due to concern about a distressing experience with food (e.g. chocking or vomiting), or due to low interest in eating (e.g. picky eating).

### Healthy eating and orthorexia nervosa

Over recent decades, healthy eating has become increasingly idealised in our society, with the focus on what, when and how much to eat becoming a fundamental part of social discourse, and often accompanied by moral judgment [11]. Statements concerning the potential dangers and health benefits of foods are now widely disseminated online. Since its launch, over 600 million people have joined Instagram, with food photos becoming increasingly popular [12] and “#food” being recognised as one of the top 25 hash tags [13]. Around half of Instagram users report using this medium to share food experiences, with 42% reporting that they also seek advice about food online [14]. However, the wide dissemination of multiple and contradictory messages about food can be confusing, with assertions about eliminating entire food groups often circulating without empirical support [15].

Because healthy eating and healthy lifestyles are viewed as desirable among society [16] and are gaining importance, it is difficult to recognise when healthy eating becomes obsessional and problematic. Currently, there is mixed information around what we should and should not eat, and what is and is not healthy [11]. This can lead to anxiety around food choices, and leads some people to search for accurate up to date food information, sometimes from less than credible sources [11]. For some individuals, this results in cutting out food groups from their diet, due to the potential harmful risks. If restrictions continue and become obsessive, this can lead to orthorexic tendencies [1, 5], which may be harmful for the individual, particularly if accompanied by co-occurring clinical symptoms such as impairments in social functioning [5]. However, not all healthy eating will become obsessional and it is important to understand associated factors that may precipitate ON.

### Diagnosis

Currently, ON is not a recognised eating and feeding disorder in the DSM-5 [2] or ICD-11 [17]. However, there is recognition from clinicians that ON exists in practice. In one study, approximately two thirds of Dutch-speaking eating disorder professionals surveyed (*n* = 111), reported that they had seen ON cases in their work and thought that ON warranted more attention both clinically and in research [18]. In a more recent study, 95.6% of eating disorder specialists surveyed in the Netherlands (*n* = 160) stated that ON was prevalent to some extent in the general population, with the majority reporting that ON should be categorised under the Eating and Feeding Disorders category in the DSM-5 [19]^2^. At present, ON would perhaps be diagnosed as a subtype of AN, due to overlapping features between ON and AN, such as perfectionism, guilt over food transgression, trait anxiety, cognitive rigidity [20].

The main difficulty in categorising ON is the lack of proper diagnostic criteria and appropriate measurement tools. Indeed, while the existing proposed classification systems broadly agree that ON involves an obsessional preoccupation with ‘healthy’, ‘pure’ or ‘clean’ foods, as well as rigid avoidance of foods considered ‘unhealthy’ or ‘unclean’, several sets of diagnostic and psychometric tools have been suggested. The ORTO-15 [21] is the most widely used psychometric tool in ON research to date [22], having been translated into several languages including Polish, German, Spanish and Hungarian [23]. This 15 item scale adapted items from the original ON screening tool, the Bratman’s Orthorexia Test [24].

#### Note: 2

Whilst many papers suggest a similarity between ON and other eating disorders, some also suggest links with obsessive compulsive disorders (OCD). Please see [6, 20, 25] for a discussion of the relationship between ON and OCD.

Several scales have been developed from it, such as the ORTO-11 [26] ORTO-11-HU [27], ORTO-9-GE [28], and ORTO-7 [29], some of these scales also using a corrected scoring (called CS) for items 1 and 13 (see 28 for a detailed explanation about the different scoring systems and scales). However, the validity and reliability of this measure and its adaptations has been questioned, and it has been found to have low internal consistency, and limited content validity [30].

More recently, other questionnaires such as the Eating Habits Questionnaire (EHQ) [31], the Düsseldorf Orthorexie Scale, DOS, [32], or the Teruel Orthorexia Scale, TOS, [23, 33] have been developed but they lack generalizability and also suffer from psychometric limitations [23, 34]. Importantly, while some studies have offered some diagnosis criteria [5, 32, 35] an official set of criteria does not yet exist [22]. Therefore, and while the ORTO-15 is not a *diagnosis* tool, it was the most suitable tool to measure orthorexic tendencies at the time the study was conducted. As a result, we refer to orthorexic tendencies in this current manuscript, and do not imply a diagnosis of orthorexia nervosa in our participants.

### Emotions

Individuals with eating disorders show difficulties identifying and describing their feelings [36] as well as regulating them [9, 10]. Research suggests that difficulties with emotions could be both a risk and maintenance factor for eating disorders [37–39]. As such, eating disorder behaviours such as bingeing, purging, or food restriction may be used as a maladaptive coping mechanism to regulate negative affect. For instance, in response to negative affect, individuals with an eating disorder tend to change their food intake, such that individuals with AN have a tendency to restrict their eating, while individuals with BN tend to eat more [40]. A reduction in negative affect after engaging in these behaviours reinforces these maladaptive strategies, which is thought to maintain the eating disorder [41]. While very little work has been conducted in ON, similar mechanisms may be in place. Indeed, in a recent qualitative study of 15 women bloggers who self-identified as having ON [42], restrictive dietary rules around healthy eating was described as a coping strategy in order to feel in control.

Eating disorders have also be defined as disorders of over-control or under-control of socio-emotional behaviours, for anorexia nervosa and bulimia nervosa respectively [43, 44]. As such, disorders of over-control in particular have been linked to social isolation, cognitive rigidity, high detailed focused processing, strong needs for structure, and hyper-perfectionism [43] traits that have also been found in individuals with ON tendencies [20]. It is therefore possible that individuals with ON tendencies also suffer from deficits in emotional processing and regulation, and that orthorexic behaviours are used as a way of regaining control.

Though difficulties with emotion regulation have been reliably associated with eating disorders, the link with alexithymia is less clear cut. Indeed, while it is estimated that as many as 77% of female patients with AN have alexithymia^3^ [45], and while this relationship is maintained even after controlling for nutritional status [46], findings are mixed as to whether alexithymia remains after controlling for general depression and anxiety, in both AN and BN (see 7 for review). Because being able to accurately identify emotions is an important first step to being able to regulate them [47, 48], exploring alexithymia is required to fully understand possible emotional processing deficits in individuals with orthorexic tendencies.

#### Note 3:

It should be noted that alexithymia is not a disorder in diagnostic manuals (DSM-5 or ICD-11), but is instead a personality trait, normally distributed in the general population, and usually associated with a range of psychopathologies [48].

### Current study

Emotional functioning in people with orthorexic tendencies has yet to be explored but could help better understand this pathology. The current study aims to address this gap by exploring whether people with orthorexic tendencies also have difficulties identifying and regulating their emotions, in order to better understand and classify ON, such as whether it should be classified as an eating disorder. Based on the literature we expected that ON tendencies would be positively associated with alexithymia and emotion regulation difficulties. Because disordered eating habits have been reliably associated with greater ON tendencies [49], we controlled for this using the Eating Attitude Test (EAT-26). We did not however have any prediction on whether the effects would remain after controlling for general eating psychopathology, given the poor psychometrics properties such as low specificity of the ORTO-15.

## Method

### Aim, design and setting of the study

#### Participants

A total of 196 participants (167 females, 29 males, mean age = 27.9, age range: 18–66) were recruited through convenience sampling on social media sites (Facebook) as well as through Bournemouth University^4^. Participants were entered in a prize draw to win two £50 Amazon vouchers. This study received ethical approval from Bournemouth University.

#### Materials

##### Orthorexia nervosa symptomatology

To measure orthorexia tendencies the ORTO-15 was used. This scale is a 15-item scale with a 4-point Likert scale from ‘always’ to ‘never’, with low scores corresponding to stronger symptoms. Example questions include: “Are your eating choices conditioned by your worry about your health status?”, “In the last 3 months, did the thought of food worry you?”, and “Do you think that the conviction to eat only healthy food increases self-esteem?”. The original scoring suggests that items 3, 4, 6, 7, 10,11, 12, 14, 15 be scored 1 = always, 2 = often, 3 = sometimes, and 4 = never. Items 2,5,8,9 should be reversed scored; and two items [1, 13] should be scored 2 = always, 4 = often, 3 = sometimes, and 1 = never. However, the scoring for the last two items has been challenged and alternate corrected scoring (CS) versions have been developed [50] using the standardised scoring procedure (i.e., 1 = always, 2 = often, 3 = sometimes, 4 = never).

Note 4: Participants were a mix of students and non-student from the UK. The participants’ occupation or ethnicity was not recorded.

The reliability of the ORTO-15 has been questioned [30] and indeed, a reliability analysis confirmed that it was very poor in our sample (α = .264). We tested the reliability of the other scales developed and as per Table 1, the ORTO-7CS is the only one reaching an acceptable alpha in our sample (α = .703) and is therefore the scale that was used in the current study.

**Table 1:** Cronbach α for the various version of the ORTO-15

Model	Item removed	Cronbach α in our sample
ORTO-15	-	.264
ORTO-15CS	-	.591
ORTO-11	1, 2, 9, 15	.542
ORTO-11CS	1, 2, 9, 15	.683
ORTO-9	1, 2, 8, 9, 13, 14	.664
ORTO-7CS	2, 5, 6, 8, 14, 15	.703

##### Emotion regulation

The Difficulty in Emotion Regulation Scale, DERS-16 [51] is a 16-item scale assessing emotion regulation. This measure uses a 5-point Likert scale with answers ranging from ‘Almost Always’ to ‘Almost Never’. Within the scale are five subscales: clarity (2 items, e.g. “I have difficulty making sense out of my feelings”), goals (3 items, e.g. “When I am upset, I have difficulty getting work done”), impulse (3 items, e.g. “When I am upset, I become out of control”), non-acceptance (3 items, e.g. “When I am upset, I become irritated with myself for feeling that way”) and strategies (5 items, e.g. “When I am upset, I believe that there is nothing I can do to make myself feel better”). Higher scores are found to indicate difficulties with emotional regulation. The DERS-short has been shown to have good reliability [52] which was confirmed in our sample (total: α = .959, clarity: α = .914; goals: α = .886; impulse: α = .916; non-acceptance: α = .874; strategies: α = .937).

##### Alexithymia

The Toronto Alexithymia Scale, TAS-20 [53] is a 20-item scale with a 4-point Likert scale. Ratings range from ‘Strongly Disagree’ to ‘Strongly Agree.’ Participants scoring 61 or above are characterised as having Alexithymia. This questionnaire includes three subscales that measure difficulty in identifying feelings (DIF, 7 items, e.g. “I am often confused about what emotion I am feeling”), difficulty describing feelings (DDF, 5 items, e.g. “It is difficult for me to find the right words for my feelings”), and externally-oriented thinking (EOT, 8 items, e.g. “I prefer to analyze problems rather than just describe them”). The TAS-20 has been shown to have good reliability [48] which was mostly confirmed in our sample, although the externally oriented thinking subscale had a relatively low reliability score (total: α = .867, DIF: α = .861; DDF: α = .796; EOT: α = .590). This was also reported in Preece et al. [48], who recommended using this subscale with caution when examined in isolation.

##### Eating psychopathology

The Eating Attitudes Test, EAT-26 [54] measures symptoms and characteristics of eating disorders. It is composed of a 26-item questionnaire with a 6-point Likert scale with answers ranging from ‘Always’ to ‘Never’. This questionnaire has three subscales measuring dieting behaviours (diet, 13 items), bulimia and preoccupation with food (bulimia, 6 items), and oral control (oral, 7 items), with a total scores greater than 20 indicating the need for further investigation by a qualified professional. The EAT-26 has been shown to have good robust psychometric properties, including good reliability [55], which was also confirmed in our sample (α = .902).

### Procedure

This study received ethical approval from Bournemouth University ethics committee. Participants completed the four questionnaires online in the following order: ORTO-15, TAS-20, EAT-26, DERS-16. After completion (9 minute duration on average), they were given the chance to enter a prize draw to win one of two £50 amazon vouchers.

### Statistical analysis

We used SPSS version 25 to run a hierarchical regression to investigate the contributions of difficulties with alexithymia and regulation in accounting for symptoms of orthorexia nervosa while controlling for other eating psychopathologies. Our dependent variable was the ORTO-7CS. In step one of the model we entered our independent variables alexithymia (TAS-20) and emotion regulation difficulties (DERS-16). In step two, we controlled for eating psychopathologies (EAT-26), to determine whether difficulties with emotions are specific to ON characteristics, or are due to shared variance with eating psychopathologies.

We then looked at how each subscales correlated with symptoms of ON tendencies. We could not add all the subscales in the hierarchical regression because they correlate highly with each other (as can be seen in Table 4). We conducted Bonferroni corrections to account for multiple correlations, adjusting the *p* value to *p =* .005 for 11 variables.

## Results

We first tested for the validity of our assumptions. Scatterplots indicated that all our IVs (EAT, TAS and DERS) had a linear relationship with our DV (ORTO-7CS). No multicollinearity was found in the data, as VIF scores were well below five (1.28, 1.48 and 1.63 respectively for EAT, TAS and DERS) and tolerance was above 0.2 (0.78, 0.68 and 0.62 respectively). The independence of residuals was checked with a Durbin-Watson statistic which showed that residuals were independent, with a value of 1.795. Finally, the residuals were normally distributed and homoscedasticity was also met. Table 2 presents the descriptive statistics for the variables of interest.

**Table 2:** Descriptive statistics of the variables of interest

	*N*	*M (SD)*	Min-Max	Number in clinical range
ORTO-7CS	192	23.9 (4.3)	12–35	*N* = 23
EAT-26	196	12.7 (11.9)	0–75	*N* = 36
TAS-20	196	49.0 (12.5)	25–85	*N* = 35
DERS-16	70	37.3 (15.7)	16–80	NA

Legend: ORTO-7CS measures symptoms of orthorexia nervosa. EAT-26 measures eating psychopathology. TAS-20 measures alexithymia and DERS-16 measures emotion dysregulation. The number in clinical range was calculated for the ORTO-7CS (number of participants whose score on the ORTO-7CS was equal to or less than 19); EAT-26 (number of participants whose score on the EAT-26 was greater than 20); and TAS-20 (number of participants whose score on the TAS-20 was equal to or greater than 61)

### Hierarchical regression

As none of the assumptions were violated, a hierarchical multiple linear regression was conducted to predict symptoms of ON based on alexithymia and emotion dysregulation (model 1), and controlled for eating psychopathology in model 2.

As shown in Table 3, while model 1 significantly accounted for 11% of the variance of ON characteristics (*adj. R*^*2*^ = .11, *F*(1, 191) = 12.1, *p* < .001), only difficulties with emotion regulation predicted scores on the ORTO-7CS (*β* = −.369, *p* < .001). The negative slope means that higher scores on the DERS-16 (more difficulties with emotion dysregulation) were linked to lower scores on the ORTO-7CS (more orthorexic tendencies). Because alexithymia was not a significant predictor, it was removed from model 2, in which only the DERS-16 and EAT-26 scores were entered. Model 2 was a much better fit (*adj. R*^*2*^ = .44, *F* (1, 191) = 77.1, *p* < .001) and interestingly, only eating psychopathologies were a significant predictor of orthorexic tendencies (*β* = −.65, *p* < .001) while emotion dysregulation became a non-significant predictor (*β* = −.04, *p* = .544). As a note, while there was no multicollinearity between the EAT-26 and the ORTO-7CS, both were highly correlated (*r* (190) = −.67, *p* < .001).

**Table 3:** Hierarchical multiple linear regression models

		Orthorexia nervosa			
		***β***	95% CI	***R***^***2***^_***adj***_	***∆R***^***2***^
Model 1	**TAS-20**	0.62	− 0.04; 0.08	.11	.11***
	**DERS-16**	−.37***	−0.15; − 0.06		
	Model sig.	*F*(1,191) = 12.1, *p* < .001			
Model 2	**DERS-16**	−.04	−0.04; 0.02	.44	.34***
	**EAT-26**	−.65***	−0.28; − 0.19		
	Model sig.	*F*(2,191) = 77.1, *p* < .001			

Legend: Model 1 shows the contributions of alexithymia and emotion dysregulation in accounting for variance in ON. Model 2 shows the contribution of emotion dysregulation and eating psychopathology (model 2) in accounting for variance in ON

Note: β = Standardised Beta effect size, R^2^_adj_ = Model fit, ∆R^2^ = Change in R^2^_asj_, *** *p* < .001, * *p* < .05

### Correlations

We then moved beyond the prediction model to look at the contribution of each individual subscales from the TAS-20 and DERS-16.

As shown in Table 4, symptoms of ON correlated with almost all subscales of the DERS, demonstrating difficulties with emotion regulation in four domains: difficulties engaging in goal-directed behaviours when upset, impulse control difficulties when upset, non-acceptance of emotional responses, and limited access to effective emotion regulation strategies. Interestingly, lack of emotional clarity did not correlate with symptoms of orthorexia, although it correlated highly with the difficulties in identifying emotion subscale of the alexithymia questionnaire, which was significantly associated with ON symptoms. No other subscales of the alexithymia questionnaire correlated with symptoms of ON. As a note, all the correlations between the ORTO-7CS and the TAS and DERS are negative because the ORTO-7CS is scored such that low scores mean high orthorexic tendencies, while the TAS and DERS are scored such that higher scores mean more difficulties with emotions.

**Table 4:** Group comparisons

	ORTO-7CS	TAS Tot	TAS DIF	TAS DDF	TAS EOT	DERS Tot	DERS C	DERS G	DERS I	DERS NA
TAS Tot	−.15	1								
TAS DIF	−.23*	.87*	1							
TAS DDF	−.07	.86*	.66*	1						
TAS EOT	−.02	.68*	.34*	.44*	1					
DERS Tot	−.33*	.56*	.60*	.50*	.19	1				
DERS C	−.18	.69*	.72*	.62*	.27*	.63*	1			
DERS G	−.27*	.36*	.41*	.35*	.07	.89*	.46*	1		
DERS I	−.33*	.52*	.55*	.44*	.22*	.87*	.53*	.73*	1	
DERS NA	−.29*	.52*	.54*	.48*	.21*	.88*	.50*	.69*	.69*	1
DERS S	−.32*	.46*	.52*	.41*	.12	.95*	.49*	.84*	.77*	.80*

Legend: Correlation table for all the TAS-20 subscales measuring alexithymia (Tot for the total score; DIF: difficulties identifying feelings; DDF: difficulties describing feelings; EOT: externally oriented thinking) and the DERS-16 subscales measuring emotion regulation difficulties (Tot: total score; C: lack of emotional clarity; G: difficulties engaging in goal-directed behaviours; I: impulse control difficulties; S: limited access to effective emotion regulation strategies; NA: non-acceptance of emotional response)

Note: * for comparisons significant at the adjusted *p* < .005 level (for 11 correlations)

## Discussion

The aim of this study was to investigate alexithymia and emotion dysregulation in a sample of individuals with various degrees of orthorexic tendencies. We found that difficulties identifying and regulating emotions was significantly associated with symptoms of ON. In particular, we found that individuals with high ON tendencies had more difficulties identifying and accepting their feelings, and resisting impulses, engaging in goal-directed behaviours and finding the right strategies when upset compared to people with low orthorexic tendencies. We also found that when controlling for general eating psychopathologies, emotion dysregulation did not remain a significant predictor of ON traits.

### Emotion regulation

It was interesting to find that emotion regulation difficulties were associated with ON tendencies because this is consistent with previous literature on other eating disorders [9]. Using the long version of the DERS, Harrison et al. [10] found that people with AN and BN also suffered from accepting their emotions, as well as difficulties regulating impulse, engaging in goal-directed behaviours, and finding the best strategy when upset, as per our participants with high orthorexic tendencies. In regards to the impulse subscale, which specifically looks at feeling out of control, many papers have indeed found that individuals with AN and BN show greater difficulties with controlling their behaviour during times of emotional distress compared with control groups [10, 56, 57]. Moreover, in our recent qualitative work exploring women’s experiences of ON as described in their online blogs [42], restrictive dietary rules around healthy eating were described as a coping strategy to feel ‘perfect’ and in control. Exercise and food rules were described as serving the same purpose; increasing levels of perceived control, regardless of which food/exercise rule was being adhered to. As such, it is possible that people with ON tendencies tend to be more impulsive when upset, or at least perceive themselves to be more ‘out of control’ when experiencing negative emotions. Regardless of whether losing control is objectively observed or subjectively perceived, the use of very thorough and obsessive food habits could thus serve to increase levels of perceived control in order to cope with difficult feelings. This may be particularly the case if the individual does not accept their emotional reactions and believes that they do not have any other strategies to make themselves feel better, which is what was observed with the emotion regulation strategies subscales of the DERS. Similar accounts about how controlling food can serve as a coping mechanism when other areas of life feel out of control are found within the ED literature [58, 59]. This would suggest that similar mechanisms may be in place in orthorexic tendencies and other eating disorders such as AN and BN. Understanding these mechanisms further is therefore worthy of further empirical investigation. So far our results suggest that where emotion regulation is concerned, individuals with orthorexic tendencies may suffer from the same difficulties as individuals with a diagnosed eating disorder. It is however worth noting that these difficulties are not specific to eating disorders, and have been thought to be a marker of general psychopathology rather than being eating disorder-specific [57]. In any case, our results suggest that individuals with orthorexic tendencies - even when other clinical symptoms such as impairments in social functioning or other medical issues were not considered - show emotion regulation difficulties suggesting psychopathology, and highlighting the need for more research into this disorder for future classification.

### Alexithymia

Alexithymia as measured with the total score was not found to be associated with ON tendencies, and only the difficulties identifying feelings subscale was significantly associated with ON symptoms. This is surprising because the clarity subscale of the DERS-16, which is strongly associated with the DIF scale of the TAS-20, did not show a significant association with ON symptoms. Moreover, a recent review of the literature [36] showed that both the DIF and DDF subscales were associated with AN and BN with large effect sizes, suggesting that difficulties with alexithymia may differ between orthorexic tendencies and diagnosable eating disorders. While many studies find a clear relationship between eating disorder behaviours and difficulties identifying and describing feelings [8, 36], some studies have found that this relationship becomes non-significant when controlling for general distress such as anxiety and depression [60, 61]. The lack of relationship between ON symptoms and difficulties describing feelings in our sample could therefore have been due to low levels of negative affect in our sample, rather than lack of alexithymia per se. This is potentially because our participants did not reach diagnostic levels of ON, which the ORTO-15 does not measure. Another possibility may relate to the way we measured alexithymia. Self-report measures such as the TAS-20 that was used in the current study may not be reliable because of the very nature of alexithymia which may make it difficult for individuals to reflect on their emotions [36, 62]. As such, the TAS-20 may more reflect people’s *beliefs* about themselves rather than provide an accurate representation of their ability [63]. It is therefore possible that our sample did not have any *perceived* difficulties identifying and regulating their feelings, but rather *behavioural*, or real-life difficulties. This could potentially explain the discrepancies in our findings between the DIF scale of the TAS and the Clarity scale of the DERS, which ask slightly different questions about these beliefs. Other measures claim to measure more behavioural aspects of alexithymia, such as the Level of Emotional Awareness Scale, LEAS [64]. Future research may want to investigate whether ON tendencies is related to *behaviourally* measured alexithymia, as well as whether it differs in clinical ON samples.

### Limitations and future research

While our results are of considerable interest to better understand the emotional profile of individuals with ON tendencies, we relied on the ORTO-15 scale (amended as per the ORTO-7CS scale), which has been shown to have poor psychometric properties [30]. In the current study we found that the ORTO-7CS strongly correlated with eating psychopathologies as measured with the EAT-26. Whilst this might suggest a substantial crossover between symptoms of ON and eating pathology more generally [65], it could also suggest a lack of specificity of the ORTO-tool as a measurement of ON tendencies. The ORTO-7CS has been found to have better psychometric properties than the longer versions of the ORTO tool [50],which we also replicated in the current study. However, the ORTO-7CS arguably lacks specificity in capturing symptoms of ON rather than disordered eating symptoms per se. Indeed, although two of the seven scale items of the ORTO-7CS focus upon the preoccupation with healthy eating (items 4 and 11), the remaining items focus upon worries and guilt around food, and one item asks about attention to calories – a symptom arguably more relevant to AN [20]. The limitations of the ORTO measures have been acknowledged in the ON literature [23, 34, 66], however a good quality validated alternative is not yet available. Although the ORTO-15 [21], and its translated and abbreviated versions [28, 30], are the most widely used measures of ON tendencies to date, these measures were developed based on items of the Bratman Orthorexia Test (a non-validated screening tool), without reference to any proposed diagnostic criteria for ON [5, 29]. Whilst several alternative measures have recently been developed, these too have been criticised, or have not yet been validated. For example, two promising scales, The Düsseldorf Orthorexie Scale, DOS [32], and the Teruel Orthorexia Scale, TOS [23, 33] have been developed in German and Spanish respectively. However, they have limited generalisability in English-speaking samples to date [23, 66]. The Eating Habits Questionnaire, EHQ [31] is perhaps the most established alternative to the ORTO scale, measuring feelings, behaviours and cognitions related to extreme healthy eating [34]. However, whilst it is proposed to have satisfactory psychometric properties in terms of internal consistency and test-retest reliability [34], this scale has been criticised for failing to include items which capture the clinical impairment of ON [23] as well as for potential limitations with its construct validity [34]. One scale which looks promising in addressing some of the limitations of other psychometrics in this area is the Barcelona Orthorexia Scale, BOS [34]. The BOS has been developed using a three round Delphi study with input from 58 international experts in disordered eating. However, although a battery of questionnaire items has been created, so far the psychometric properties of this new scale have not yet been tested. Therefore, whilst this study relies upon the ORTO-7CS as the most psychometrically sound version of the existing ORTO measurement scales, this is limited by the potential lack of specificity in picking up on symptoms specific only to ON rather than disordered eating more generally. Results must therefore be interpreted with caution, and further research is needed to replicate these findings using a more sophisticated measurement scale, or even a proper diagnostic tool, once developed.

In addition to the already mentioned limitations regarding the measurement tools and the lack of measurement of general negative affect in this study, our results are also limited due to its cross-sectional design. It is indeed not clear whether emotion difficulties cause symptoms of ON, or whether it is a consequence. While some models of anorexia nervosa, such as the cognitive interpersonal model, suggest that socio-emotional difficulties may be both a cause and a consequence [39], more work should investigate this in ON. Another limitation lies in the fact that we did not measure past or current eating disorders in our participants, which could have had an influence on our results. Finally, our study may be limited due to the way we recruited our participants, using Facebook. We recommend future work to widen the recruitment platforms such as through using Twitter which could have helped select participants who regularly tweet about healthy eating- this may have increased the number of participants with ON tendencies, and potentially even the severity of their symptoms. Future work should also consider conducting more qualitative studies to better understand the aetiology of ON and develop better tools to measure this disorder.

## Conclusions

In conclusion, the present research suggests that people with high orthorexic tendencies have difficulties identifying and regulating their emotions, similarly to other eating disorders. However, ON symptoms did not seem to be associated with difficulties describing emotions, unlike other eating disorders. We suggest that ON behaviours may be used as a coping strategy in order to feel in control in these participants who have poor emotion regulation abilities, although future research should specifically test this hypothesis. Our research also highlights the need to develop and validate better measure of ON to confirm our findings and advance this exciting area of research, and determine whether ON should be categorised as an eating disorder.

## Data Availability

The datasets used and/or analysed during the current study are available from the corresponding author on reasonable request.

## Abbreviations

DERS-16: Difficulties with Emotion Regulation Scale (16 item version)
DIF: Difficulty Identifying Feelings, a subscale of the TAS-20
DDF: Difficulty Describing Feelings, a subscale of the TAS-20
EAT-26: Eating Attitudes Test (26 item version)
ED: Eating Disorders
EOT: Externally-Oriented Thinking, a subscale of the TAS-20
ON: Orthorexia Nervosa
ORTO-15: Orthorexia nervosa questionnaire (15 item version)
ORTO-7CS: Orthorexia nervosa questionnaire (7 item version correct score)
TAS-20: Toronto Alexithymia Scale (20 item version)
